# Does baseline [18F] FDG-PET/CT correlate with tumor staging, response after neoadjuvant chemoradiotherapy, and prognosis in patients with rectal cancer?

**DOI:** 10.1186/s13014-018-1154-3

**Published:** 2018-10-25

**Authors:** Letizia Deantonio, Angela Caroli, Erinda Puta, Daniela Ferrante, Francesco Apicella, Lucia Turri, Gianmauro Sacchetti, Marco Brambilla, Marco Krengli

**Affiliations:** 1 Radiotherapy, University Hospital “Maggiore della Carità”, Novara, Italy; 2 Department of Translational Medicine, University of “Piemonte Orientale”, Novara, Italy; 3 Nuclear Medicine, University Hospital “Maggiore della Carità”, Novara, Italy; 4 Department of Translational Medicine, Unit of Medical Statistics and Cancer Epidemiology, CPO Piemonte and University of “Piemonte Orientale”, Novara, Italy; 5 Medical Physics, University Hospital “Maggiore della Carità”, Novara, Italy

**Keywords:** Rectal cancer, [18F] fluorodeoxyglucose positron emission tomography, Standardized uptake value, Metabolic tumor volume, Total lesion glycolysis, Predictive value

## Abstract

**Background:**

[18F] fluorodeoxyglucose positron emission tomography/computed tomography ([18F] FDG-PET/CT) may be used for tumor staging and prognosis in several tumors but its role in rectal cancer is still debated. The aim of the present study was to assess the correlation of baseline [18F] FDG-PET parameters with tumor staging, tumor response (tumor regression grade (TRG)), and outcome in a series of patients affected by locally advanced rectal cancer (LARC) treated with neoadjuvant chemoradiotherapy (CRT).

**Methods:**

One hundred patients treated with neoadjuvant CRT and radical surgery were enrolled in the present study. Maximum standardized uptake value (SUVmax), SUVmean, metabolic tumor volume (MTV), and total lesion glycolysis (TLG) at the baseline [18F] FDG-PET were calculated. These PET parameters were correlated with tumor staging, histopathological data (TRG1 vs. TRG2–5 and TRG1–2 vs. TRG3–5), disease-free survival, and overall survival.

**Results:**

SUVmax and SUVmean of primary tumor were statistically associated with T4-stage. SUVmax, SUVmean, and TLG did not result statistically associated with TRG (TRG1 or TRG1–2). MTV resulted statistically associated with TRG1–2 group (OR 2.9; 95% CI 1.2–7.1). Finally, no PET parameter was significantly associated with disease-free or overall survival.

**Conclusion:**

Our results showed that baseline [18F] FDG-PET parameters correlated with tumor staging, and only MTV correlated with TRG 1–2. PET parameters failed to predict disease-free and overall survival after treatment completion. The results leave open to further studies the issue of identifying patients suitable for conservative approaches.

## Background

Treatment approach to rectal cancer has greatly evolved over the past years. In locally advanced rectal cancer (LARC), neoadjuvant chemoradiotherapy (CRT) followed by surgical resection with total mesorectal excision is the current standard treatment approach [1, 2].

According to literature data, 50 to 60% of the patients can reach a down-staging following CRT with about 20% of complete pathological response [3]. This rate of pathological response has raised the issue of organ preservation. Notably, Habr-Gama et al. [4] pioneered this innovative concept by avoiding surgery when a complete response was achieved at restaging after CRT. The study reported 49% of complete clinical response after CRT in a series of 183 patients with T2–4 N0–2 distal rectal tumors. Subsequently, the “watch and wait” strategy or the local excision after CRT has been explored as organ-preserving alternatives in selected patients’ cohorts with encouraging results [5, 6].

Tumor response after CRT is usually assessed by the tumor regression grade (TRG) as proposed by Mandard et al. [7], based on the pathological exam of the surgical specimen. Nowadays, the diagnostic challenge for an organ preservation approach is to find an adequate surrogate of histology able to discriminate responders from non-responders. The attention on morphological (i.e. magnetic resonance imaging, MRI) and even more on metabolic imaging suitable for predicting CRT response is constantly increasing [8, 9]. In this regard, the role that [18F] -fluorodeoxyglucose positron emission tomography/computed tomography ([18F] FDG-PET/CT) has in staging and treatment planning of rectal cancer is still quite debatable [10–12] as well as in measuring treatment response [13–18].

The primary aim of the present study was to evaluate whether in LARC metabolic characteristics detected by [18F] FDG-PET/CT, prior to neoadjuvant CRT, could correlate with clinical tumor stage, with tumor regression after surgery, and with MRI findings. A secondary endpoint was to determine the prognostic value of [18F] FDG-PET/CT in terms of disease-free survival (DFS) and overall survival (OS).

## Methods

### Patients

From 2008 to 2016, one hundred patients were enrolled in the present observational study after obtaining a written informed consent following the rules of our institution. The local ethics committee approved the present study. Inclusion criteria were as follows: biopsy proven rectal adenocarcinoma, age > 18 years, cT3–4 cN0–2 disease at staging, absence of distant metastasis or other concomitant tumors, no contraindication to chemotherapy, and availability of the [18F] FDG-PET/CT images retrieved from our institutional digital archive. All cases were discussed in a multidisciplinary conference with gastroenterologists, surgeons, radiation oncologists, and medical oncologists.

Pre-treatment workup included blood chemistry, clinical examination, endoscopy with biopsy, and chest and abdomen CT. Tumor extension and lymph-nodal involvement were assessed by pelvic MRI on a 1.5 Tesla scanner (Achieva, Philips Medical System, Best, Holland) and [18F] FDG-PET/CT [8]. Final staging was defined according to the American Joint Committee on Cancer (AJCC) TNM classification [19]. The main clinical characteristics of patients are listed in Table 1.

**Table 1:** Patients’ clinical characteristics

Characteristics	Value	(%)
Gender (No. of patients)		
Male	65	(65.0)
Female	35	(35.0)
Age (years)		
Median	67	
Range	41–83	
Tumor clinical stage (No. of patients)		
cT3	85	(85.0)
cT4	15	(15.0)
Nodal clinical stage (No. of patients)		
cN0	32	(32.0)
cN+	68	(68.0)
Rectal segment, distance to the anal verge		
Upper third	20	(20.0)
Middle third	42	(42.0)
Lower third	38	(38.0)
Pathological stage (No. of patients)		
ypT0 N0	16	(16.0)
ypT1 N0-N1	5	(5.0)
ypT2 N0-N2	19	(19.0)
ypT3 N0-N1-N2	56	(56.0)
ypT4 N0-N1-N2	4	(4.0)
TRG (No. of patients)		
TRG 1	16	(16.0)
TRG 2	15	(15.0)
TRG 3	25	(25.0)
TRG 4	22	(22.0)
TRG 5	22	(22.0)

*No* number, *TRG* Tumor regression grade

### [18F] FDG-PET/CT imaging

The patients underwent [18F] FDG-PET/CT within five working days from CT simulation by hybrid PET/CT scanner (Biograph 16 HI-REZ, Siemens Medical Solutions, Berkeley CA), equipped with Pico-3D digital electronics and reconstruction with ordered subset expectation maximization (OSEM3D) algorithm (2 iterations × 24 subsets, and a post-reconstruction Gaussian filter with a full width at maximum of 8 mm), on a 256 × 256 image frame (voxel size 2.66 mm × 2.66 mm × 2.00 mm). Images were analyzed using a dedicated workstation Leonardo (Siemens Medical Solutions, Berkeley CA). CT images were used for both attenuation correction of PET data and localization of pathological FDG uptake. CT scan was performed without administration of intravenous contrast with a low-dose protocol for CT acquisition. Fasting time was at least 6 h prior to examination. The blood glucose levels of all patients were measured before the injection of [18F] FDG and were < 150 mg/dL. The injected activity of FDG was 3 MBq/Kg and the mean time between injection and acquisition was 70 min (range: 55–90 min). [18F] FDG-PET/CT imaging was performed from the proximal femur to the base of the skull. Emission images were acquired for 2–5 min per bed position, depending on patient’s body mass index as described in a previous study [20].

The processed images were displayed in coronal, transverse, and sagittal plans and interpreted in standard clinical fashion, both separately and in fused mode. The [18F] FDG-PET/CT images were reviewed for abnormal FDG uptake of the primary tumor, lymph nodes, and distant sites. The maximum standardized uptake value (SUVmax) was calculated for each primary tumor, along with the mean standardized uptake value (SUVmean), the metabolic tumor volume (MTV) and the total lesion glycolysis (TLG) according to the European Agency of Nuclear Medicine (EANM) guidelines [21].

The SUVmax was automatically calculated to determine the [18F] FDG-PET activity and recorded using a volumetric region of interest (VOI), positioned around the pathological [18F] FDG uptake in the attenuation-corrected images. Each VOI was checked visually to exclude areas of physiological uptake as bladder. The MTV was defined as the volume of hypermetabolic tissue with a threshold greater than 41% of the maximum pixel value in the primary tumor [21]. The software calculated the SUVmean within the MTV. The TLG was defined as the SUVmean multiplied by the MTV.

### Treatment

All patients were treated with neoadjuvant CRT. External beam RT was delivered by Linear accelerators (Varian Clinac 600 DBX and Varian Clinac DHX, Varian Medical Systems, Milpitas CA) to a total dose of 45 Gy, with daily fraction of 1.8 Gy. RT was given to all patients in a homogeneous way including the identification of the clinical target volume (CTV) which was defined as the gross tumor volume with the mesorectal fascia and the pelvic lymph nodes as recommended by Roels et al. [22]. An isotropic expansion of 8 mm around the CTV was applied to define the planning target volume (PTV). Treatment planning (Pinnacle, Philips, Adac Laboratories, Milpitas CA) was performed through 3-dimension-conformal RT (3D-CRT) technique in 57 patients and by “step and shot” intensity modulated radiotherapy (IMRT) in 43 patients. In the present study, [18]F FDG-PET/CT imaging was used for both tumor staging, and target identification as reported in a previous study [8]. Concomitant chemotherapy was administered during the five weeks of RT with daily oral capecitabine (825 mg/m^2^ twice daily) or continuous infusion of 5-fluorouracil (225 mg/m^2^).

After CRT, all patients were re-staged with the same imaging modality (CT/MRI) performed for baseline tumor staging, in particular 42 patients were re-staged with MRI (TSE T2 weighted and post-contrast weighted TFE T1 sequences). A total mesorectal excision with anterior or abdominoperineal resection was performed 6–11 weeks (median 8 weeks) after CRT according to the clinical presentation.

### Clinical endpoints

Data was collected to evaluate metabolic parameters detected by [18F] FDG-PET/CT prior to neoadjuvant CRT in relation with pre-treatment tumor characteristics (i.e. tumor extension, distance from the anal verge, and presence of pathological lymph-nodes), treatment response, response assessment with MRI, and survival.

Post-surgical staging was assessed according to the AJCC/TNM classification (ypTNM) [19]. A pathological finding of ypT0N0 was considered a complete pathological response, while any ypT1–4 N0–2 a non-complete pathological response (Table 1). These findings were scored according to TRG classification following the criteria defined by Mandard et al. [7]: TRG1 = complete tumor response, no residual cancer cell; TRG2 = residual cancer cells scattered through fibrosis; TRG3 = increased number of residual cancer cells with predominant fibrosis; TRG4 = residual cancer outgrowing fibrosis; and TRG5 = no regressive changes within the tumor. We performed the analysis between TRG1 and TRG2–5 and between TRG1–2 and TRG3–5.

In the subset of 42 patients who repeated MRI after neoadjuvant treatment (TSE T2 weighted, post-contrast weighted TFE T1 sequences), the response assessment, scored by Response Evaluation Criteria in Solid Tumors (RECIST) [23] was compared with PET parameters.

During follow-up, patients underwent the same exams as at baseline every 6 months for the first 2 years and then every year. Diagnosis of recurrent tumor and distant metastasis was based on clinical and radiological evidence of tumor relapse, confirmed by biopsy.

### Statistical analysis

All quantitative PET imaging parameters (SUVmax, SUVmean, MTV, and TLG) were expressed as mean ± standard deviation (SD). The student 2-tailed t-test after normalization using logarithmic transformation was used to compare the quantitative parameters of the clinical variables categorized as follows: cT3 vs. cT4, TRG1 vs. TRG2–5, and TRG1–2 vs. TRG3–5.

Binary logistic regression was performed to evaluate the association of SUV, MTV and TLG with TRG using the median value as cut-off. The odds ratios (OR) and the 95% confidence interval (95% CI) were calculated. The analysis of variance (ANOVA) after normalization using logarithmic transformation was performed to evaluate PET parameters differences among upper, middle and lower rectal cancer locations. Mann Whitney test was performed to analyze the time interval from the end of CRT and surgery among TRG classes.

Logistic regression was applied to analyze and compare PET and MRI images at restaging.

Follow-up time was analyzed from the last day of CRT to the date of the last follow-up, recurrence, or death. DFS and OS rates were calculated using Kaplan-Meier analysis stratified according to cut-off value (median value) and compared using the log-rank test. Recurrent and non-recurrent patients were compared with Fisher test.

The statistical power of the study was 50%. This was calculated on 100 patients enrolled, on value of alpha equal to 0.05, and on the 4-years OS for SUVmax median value as cut-off equal to 0.90 (for patients with a SUVmax < 20.6) and to 0.75 (for patients with a SUVmax > 20.6). A *p* value ≤0.05 was considered as statistically significant. Statistical analysis was performed using STATA v11 (Stata Corporation, College Station, TX, USA).

## Results

### Metabolic characteristics in relation to clinical stage and restaging

The mean values and SD of SUVmax, SUVmean, MTV, and TLG of the baseline [18F] FDG-PET/CT were reported in Table 2. The SUVmax (*p* = 0.01), SUVmean (*p* = 0.01), MTV (*p* = 0.003) and TLG (*p* = 0.0004) values were significantly higher in the cT4 than in the cT3 cases. Conversely, PET parameters were not influenced by rectal tumor origin in the upper, middle and lower rectum. By logistic regression model, primary tumors with SUVmax or SUVmean higher than median values were statistically associated with cT4-stage (both ORs 8.4; 95% CI 1.8–39.7).

**Table 2:** [18F] FGD-PET parameters of the primary tumor

Variable	Mean (SD)	Minimum	Maximum
SUVmax	22.7 (9.7)	5.7	54.1
SUVmean	13.2 (5.7)	3.1	32.6
MTV	21.4 (21.9)	1.2	153.9
TLG	313.0 (480.0)	8.0	3331.0

*SD* Standard deviation, *SUVmax* Maximum standardized uptake value, *SUVmean* Mean standardized uptake value, *MTV* Metabolic tumor volume, *TLG* Total lesion glycolysis

Response assessment with MRI was available in 42 patients out of 100. None of the PET parameters resulted statistically associated with MRI findings (1 complete response and 20 partial responses vs. 11 stable diseases).

### Pathological response evaluation

All patients underwent surgery with total mesorectal excision 6–8 weeks after CRT; anterior resection was performed in 79 patients (79%) and abdominoperineal resection in 21 patients (21%). The ypTNM classification was available for all the 100 patients (Table 1). At pathological examination, 16 patients (16%) were classified as TRG1 (ypT0 N0) and 84 (84%) as TRG2–5 (ypT1–4 N0–2). Sixty-one out of 100 patients (61%) achieved tumor downstaging. Negative margins were observed in all but two patients, classified as TRG3 and TRG5 respectively, after neoadjuvant CRT.

Comparing [18F] FDG-PET parameters of TRG1 and TRG2–5 no significant difference was found, but comparing TRG1–2 and TRG3–5 MTV significantly changed (14.8 vs. 24.4, *p* = 0.01). Thus, only MTV resulted statistically associated with TRG1–2 group (OR 2.9; 95% CI 1.2–7.1) (Table 3).

**Table 3:** [18F] FGD-PET/CT parameters related to clinical variables

Variables	cT stage Mean ± SD		TRG Mean ± SD		TRG Mean ± SD	
	cT3	cT4	TRG1	TRG2–5	TRG1–2	TRG3–5
SUVmax	21.6 ± 9.3	28.9 ±9.8	22.9 ±9.9	21.7 ±8.8	22.9 ±8.9	21.7 ± 10
	*p* = 0.01		*p* = 0.68		*p* = 0.75	
SUVmean	12.6 ± 5.5	16.6 ± 5.7	13.3 ± 5.9	12.5 ± 4.9	13.5 ± 5.4	13.3 ± 5.9
	*p* = 0.01		*p* = 0.68		*p* = 0.6	
MTV	18.0 ± 13.2	41.0 ± 43.2	22.4 ± 23.3	16.7 ± 11.7	14.8 ± 10.3	24.4 ± 24.9
	*p* = 0.003		*p* = 0.21		*p* = 0.01	
TLG	221.1 ± 172.6	833.5 ± 1053.0	330.1 ± 515.4	222.9 ± 200.8	210.6 ± 183.9	358.9 ± 560
	*p* = 0.0004		*p* = 0.23		*p* = 0.08	

*T* Tumor stage, *TRG* Tumor regression grade, *SD* Standard deviation, *SUVmax* Maximum standardized uptake value, *SUVmean* Mean standardized uptake value, *MTV* Metabolic tumor volume, *TLG* Total lesion glycolysis, *p* p- value calculated with Student T-test

Analyzing interval time between the last day of CRT and surgery, no significant differences were observed between TRG1 and TRG2–5 (*p* = 0.49) or TRG1–2 and TRG3–5 (*p* = 0.30).

### Metabolic characteristics in relation to DFS and OS

**Fig. 1:**
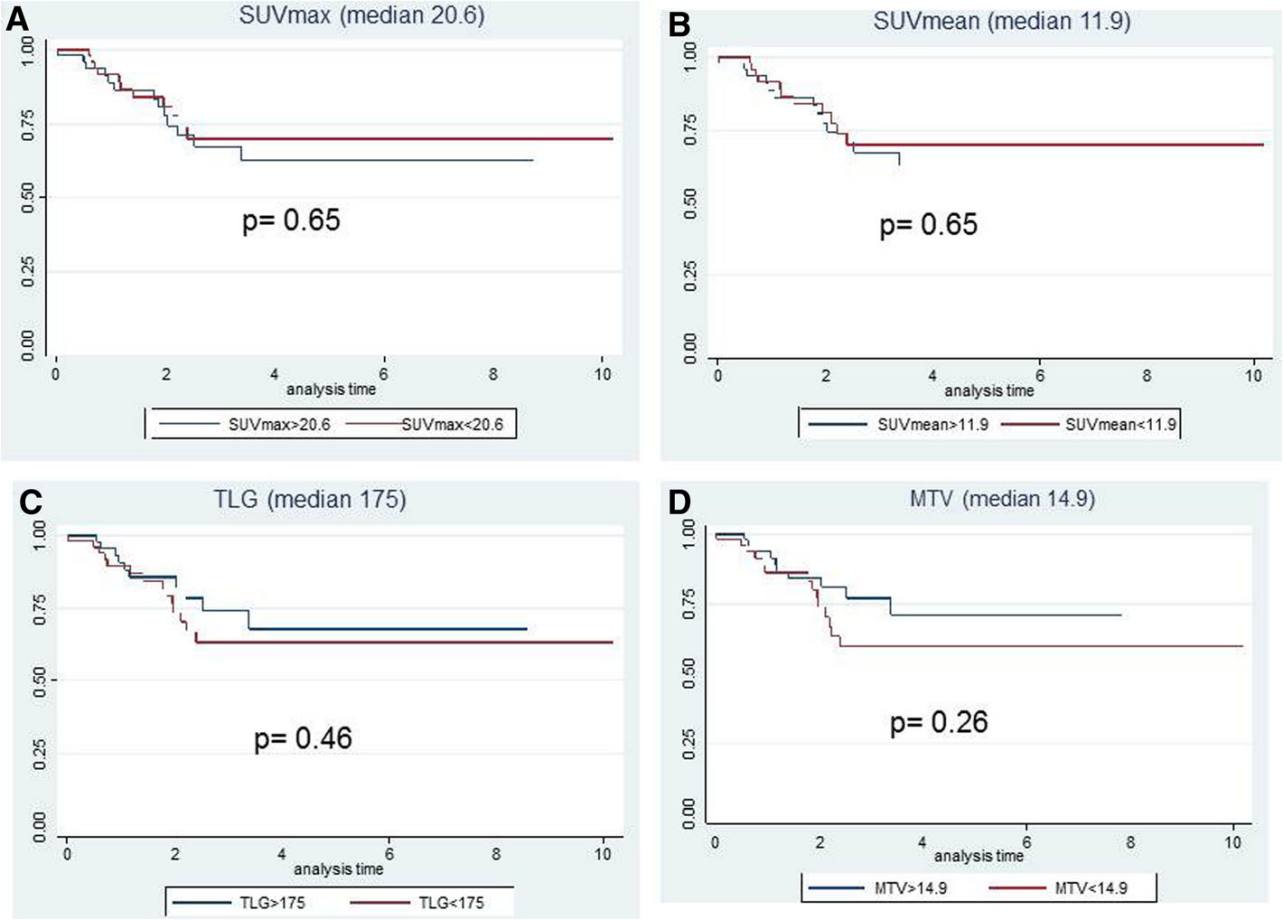
4-years disease-free survival (DFS). Kaplan-Meier curves with the Log-Rank value of the PET parameters (SUVmax, SUVmean, TLG, and MTV) using the median values as cut-off. The blue lines correspond to values higher than median, the red lines correspond to values lower than the median

**Fig. 2:**
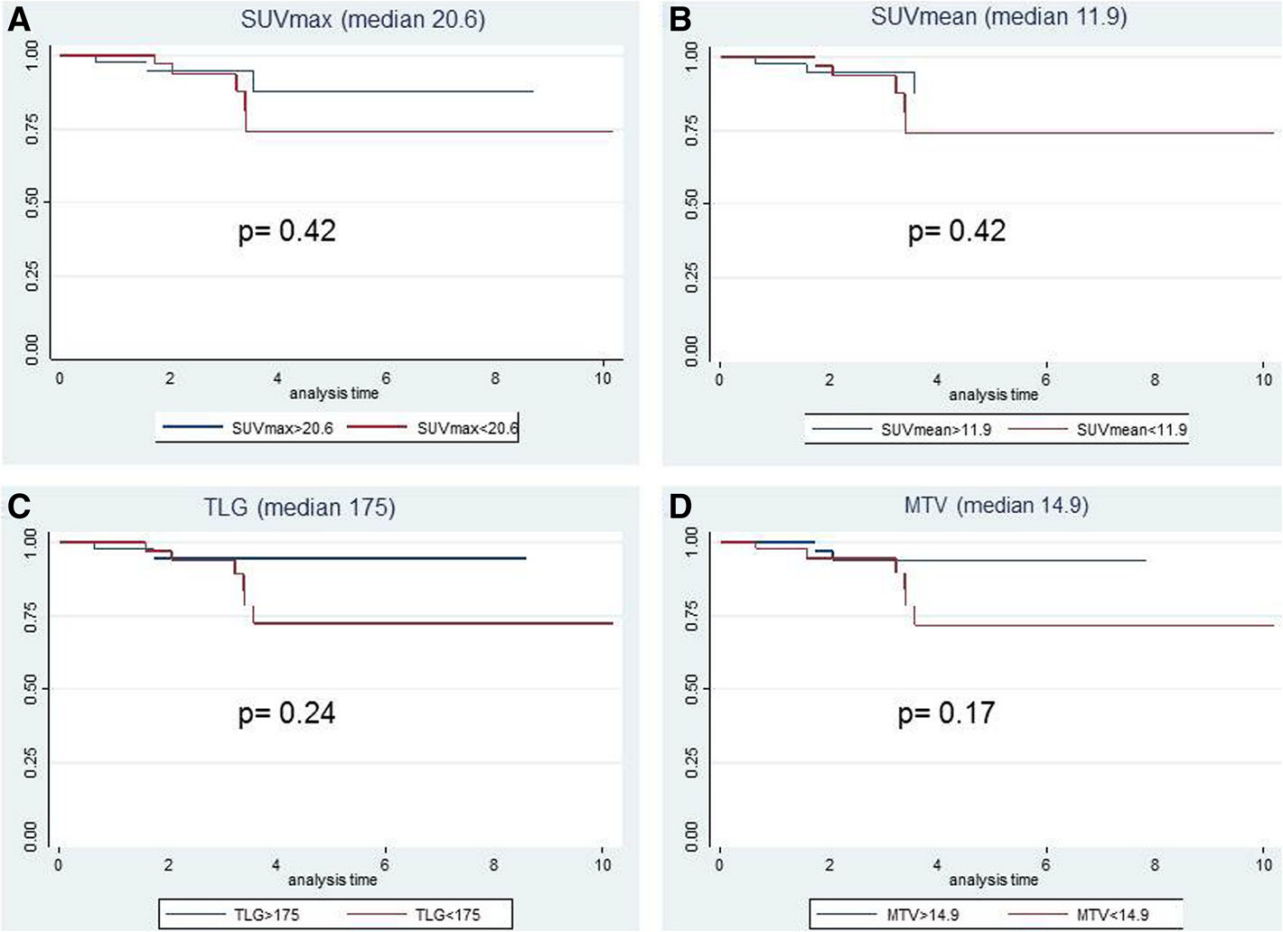
4-years overall survival (OS). Kaplan-Meier curves with the Log-Rank value of the PET parameters (SUVmax, SUVmean, TLG, and MTV) using the median values as cut-off. The blue lines correspond to values higher than median, the red lines correspond to values lower than the median

The median follow-up time of the whole series was 32 months (range 12–116 months). Twenty-four patients (24%) developed loco-regional or at distance relapse. Although all but one of them were classified as TRG2–5 at the histological analysis, the comparison of tumor relapses between TRG1 and TRG2–5 did not achieve a statistical significance (*p* = 0.1). Ten patients developed a loco-regional recurrence (41.7%) and 14 liver and/or lung metastasis (58.3%). At the time of analysis, 8 patients were dead (8%), 6 out of the 8 died of metastatic progression disease. Moreover, none PET parameters significantly correlated with DFS or OS (Figs. 1 and 2).

## Discussion

It is well recognized that neoadjuvant CRT for LARC has an impact on tumor downsizing and can achieve a higher percentage of long-term local control and better quality of life than adjuvant CRT [24]. Moreover, the achievement of a complete pathological response has a demonstrated positive prognostic impact on disease control [25]. Nowadays, the challenge is how to discriminate responders from non-responders to preoperative CRT, in order to tailor the treatment strategy. Less aggressive surgery or “watch and wait” policy after CRT for responders or more aggressive treatment for non-responders. In this setting, the most appropriate imaging approach to assess tumor response is a key point and it is still matter of investigation. In this regard, endorectal ultrasounds and MRI are routinely used for re-staging before surgical procedure, but these imaging modalities suffer of limited accuracy in distinguishing residual tumor from post-treatment changes such as fibrosis [26]. Of note, some authors [27–30] investigated the early prediction of tumor response based on [18F] FDG-PET/CT imaging performed during and after neoadjuvant CRT by comparing the baseline [18F] FDG-PET with that ad interim and before surgery. They observed that tumor response was predicted by metabolic changes in the first two weeks of RT, proving that [18F] FDG-PET/CT can be useful to predict pathological response for rectal cancer. A weakness of these studies [27–30] was related to the small sample size of patients that could have biased the results.

Our observational study analyzed whether the only pretreatment [18F] FDG PET/CT could be correlated with tumor stage and predict tumor response and survival in a relatively large number of patients. Because the choice of the best PET parameter to be used is still debatable, in addition to the most frequently used parameters (i.e. SUVmax and SUVmean), we decided to investigate also MTV and TLG which are be able to predict treatment response in other tumor types [31, 32].

In our study, a higher level of metabolic and volumetric parameters was significantly associated with cT4 stage, generally considered a more aggressive disease.

Although the median baseline SUV, MTV, and TLG showed higher values in TRG2–5 than TRG1, the absence of a significant difference seems to support the conclusion that the baseline [18F] FDG-PET parameters cannot predict the achievement of rectal cancer complete regression after neoadjuvant CRT. We also considered PET parameters in the light of MRI response assessment in a subset of 42 patients and we were not able to find any significant association with tumor response detected by MRI performed before surgery. In this regard, a future study could consider the use of PET/MRI, including also DWI sequences, in the attempt to optimize the response assessment.

Exploring the hypothesis that baseline[18F] FDG-PET parameters might be able to predict patients with a complete pathologic response, who may be treated with organ preserving strategies, we compared either TRG1 vs. TRG2–5 or TRG1–2 vs. TRG3–5. As a matter of fact, only TRG1 cases represent true complete responses in which a transanal endoscopic resection or a wait and see policy could be justified. However, MTV resulted predictive of tumor response only when TRG1–2 was compared to TRG3–5. In this regard, only few studies [26, 33–35] analyzed the predictive value of baseline [18F] FDG-PET parameters with contrasting results, leaving this issue still open for further investigation. Chennupati et al. [33] did not find any correlation between pathological response and SUVmax or MTV at baseline in 35 patients who underwent [18F] FDG-PET. Similar findings on 88 patients were reported by Park el al. [26] who found no differences of the pre-treatment [18F] FDG-PET parameters (SUV or MTV) between responder and non-responder groups. In contrast, Bang et al. [34] in a sample of 74 patients found that MTV calculated using different thresholds was significantly associated with TRG1–2, however this association was not confirmed after multivariate analysis. Moreover, Hatt et al. [35] found that response to CRT in a small sample of 28 patients was correlated with higher levels of SUVmean (*p* = 0.02) but not with TLG and MTV.

Considering the survival findings, we have not observed any significant correlation of [18F] FDG-PET parameters with DFS or OS (Figs. 1, and 2). However, all but one patient, who experienced a loco-regional or at distance progression, were observed in the non-responder group. Most likely, the low number of tumor relapses could have influenced the non-significance of the correlation of PET parameters with survival. In this regard, literature studies reported only some and contrasting data on the correlation between PET parameters and survival. Bang et al. [34] reported that baseline MTV calculated with various thresholds was significantly associated with 3-year DFS, while Leccisotti et al. [36], analyzing baseline post-treatment metabolic changes, did not find any correlation with DFS or OS because of the very low rate of tumor relapses after a median follow-up time of 68 months.

As shown by our study and by the other literature series, the predictive role of [18] FDG-PET is still unclear and deserves further investigation (Table 4). Although the present study has the limitation of a relatively low statistical power, most likely related to the low number of tumor relapses, it analyzed quite a large number of patients treated in a homogeneous way at the same institution and observed for quite a long follow-up time.

**Table 4:** Comparison among principal clinical series and our series

Study	Number of patients	Pathological responders % (TRG)	PET parameter	Correlation PET with pCR *p Value*	Correlation PET with DFS/OS
Martoni 2011 [15]	80	20% (TRG4 by Dworak)	SUVmax pre SUVmax post	Negative *p* = 0.05 Negative *p* = 0.0003	Disease recurrence: SUVmax post >/< 5 p = 0.0003
Calvo 2013 [16]	38	50% (TRG3–4 by Dworak)	SUVmax pre SUVmax post	Negative *p* = 0.12 Negative *p* < 0.0001	ΔSUVmax< 4 risk of Recurrence *p* = 0.0007 ΔSUVmax< 4 HR = 5.73 *p* = 0.05
Park 2014 [26]	88	19.3% (TRG1 by Mandard)	SUVmax pre SUVmax post MTV pre MTV post	NS *p* < 0.001 *p* = 0.029 *p* < 0.0001	NA
Dos Anjos 2016 [28]	90	22.2% (TRG NA)	ΔSUVmax (40%) ΔMTV (40%) ΔTLG (40%)	p < 0.0001 *p* = 0.005 *p* = 0.03	NA
Janssen 2012 [30]	26 20	42.3%(TRG1–2 by Mandard) 20% (TRG1–2 by Mandard)	RI SUVmax	48% SUVmax: Spec 100% Sens 64% Spec 93% Sens 83%	NA
Chennupati 2012 [33]	35	40% (TRG 0–1 by Ryan)	SUVmax pre MTV pre SUVmax post MTV post ΔSUVmax ΔMTV	NS NS NS NS NS NS	NA
Bang 2016 [34]	74	23% (TRG 0–1 by AJCC)	SUVmax pre SUVpeak pre SUVmean pre + 2 SDs	NS NS negative *p* = 0.0045	3-years DFS NS 3-years DFS NS 3-years DFS *p* = 0.01
Leccisotti 2015 [36]	126	24.6% (TRG1 by Mandard)	Early RI SUVmax Late RI SUVmax	Cut-off 61% *p* < 0.001 cut-off not found	10.3% local recurrences 4.8% deaths
Our series	100	16% (TRG1 by Mandard) 31% (TRG1–2 by Mandard)	SUVmax pre SUVmean pre MTV pre TLG pre SUVmax pre SUVmean pre MTV pre TLG pre	NS NS NS NS NS NS p = 0.01 NS	4-years DFS NS 4-years OS NS

*pCR* Pathological complete response, *TRG* Tumor regression grade, *RI* Response index, *Spec* Specificity, *Sens* sensibility, Δ indicates percent residual, *pre* Before radiochemotherapy, *post* After radiochemotherapy, *SD* Standard deviation, *SUVmax* Maximum standardized uptake value, *SUVmean* Mean standardized uptake value, *MTV* Metabolic tumor volume, *TLG* Total lesion glycolysis, *AJCC* American Joint Committee on Cancer, *NS* Not significant, *NA* Not available

## Conclusions

This study describes one of the largest series analyzing baseline [18] FDG-PET parameters in LARC. The results showed that SUVmax and SUVmean correlated with tumor staging at diagnosis, and MTV was predictive of tumor response. However, no parameter was predictive of true complete response (TRG1). Moreover, none of the analyzed parameters was able to predict DFS and OS.

The issue of identifying patients suitable for conservative approaches is still open, and different schedules of imaging studies, including repeated PET after neoadjuvant CRT, should be explored more deeply in the view of a true personalized treatment modality.

## Abbreviations

(SUVmean): Mean standardized uptake value
[18F] FDG-PET/CT: [18F] fluorodeoxyglucose positron emission tomography/computed tomography
3D-CRT: 3-dimension-conformal RT
AJCC: American Joint Committee on Cancer
ANOVA: Analysis of variance
CI: Confident interval
CRT: Chemoradiotherapy
CTV: Clinical target volume
DFS: Disease-free survival
EANM: European Agency of Nuclear Medicine
IMRT: Intensity modulated radiation therapy
LARC: Locally advanced rectal cancer
MRI: Magnetic resonance imaging
MTV: Metabolic tumor volume
OR: Odd ratio
OS: Overall survival
PTV: Planning target volume
RECIST: Response evaluation criteria in solid tumors
SD: Standard deviation
SUVmax: Maximum standardized uptake value
TLG: Total lesion glycolysis
TRG: Tumor regression grade
